# Supplementary material for: Vibrational Spectral Analysis of Natisite (Na2TiSiO5) and its Structure Evolution in Water and Sulfuric Acid Solutions
Source: Materials (Basel). 2021 Apr 27;14(9):2259. doi: 10.3390/ma14092259 (PMC8123781; doi:10.3390/ma14092259)
Supplement: Supplementary file 1 [file materials-14-02259-s001.zip › materials-1157960-Supplementary Materials.pdf]

materials

MDPI

Supplementary Materials

Vibrational Spectral Analysis of Natisite (Na_2TiSiO_5) and its Structure Evolution in Water and Sulfuric Acid Solutions

Fancheng Meng [1,2], Yahui Liu [1], Lina Wang [1,*], Desheng Chen [1], Hongxin Zhao [1], Yulan Zhen [1], Jing Chen [3] and Tao Qi [1]

[1] National Engineering Laboratory for Hydrometallurgical Cleaner Production Technology, Institute of Process Engineering, Chinese Academy of Sciences, Beijing 100190, China; fcmeng@ipe.ac.cn (F.M.); yhliu@ipe.ac.cn (Y.L.); dshchen@ipe.ac.cn (D.C.); hxzhao@ipe.ac.cn (H.Z.); ylzhen@ipe.ac.cn (Y.Z.); tqi@ipe.ac.cn (T.Q.)

[2] State Key Laboratory of Complex Nonferrous Metal Resources Clean Utilization, Kunming University of Science and Technology, Kunming 650093, China

[3] Institute of Nuclear and New Energy Technology, Tsinghua University, Beijing 100084, China; jingxia@tsinghua.edu.cn

* Correspondence: linawang@ipe.ac.cn; Tel.: +86-10-62584427

Citation: Meng, F.; Liu, Y.; Wang, L.; Chen, D.; Zhao, H.; Zhen, Y.; Chen, J.; Qi, T. Vibrational Spectral Analysis of Natisite (Na_2TiSiO_5) and its Structure Evolution in Water and Sulfuric Acid Solutions. *Materials* **2021**, *14*, 2259. https://doi.org/10.3390/ma14092259

Publisher's Note: MDPI stays neutral with regard to jurisdictional claims in published maps and institutional affiliations.

Copyright: © 2021 by the authors. Licensee MDPI, Basel, Switzerland. This article is an open access article distributed under the terms and conditions of the Creative Commons Attribution (CC BY) license (http://creativecommons.org/licenses/by/4.0/).

1. Figures

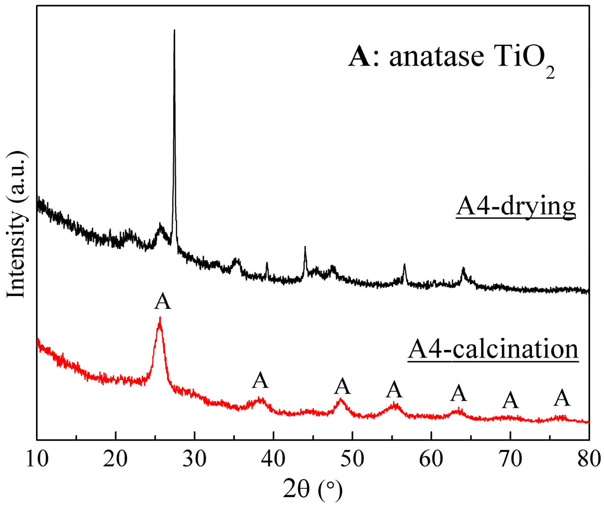

Figure S1. XRD pattern of the calcined product at 700 °C of the solid sample A4 from washing natisite with water and sulfuric acid solutions.

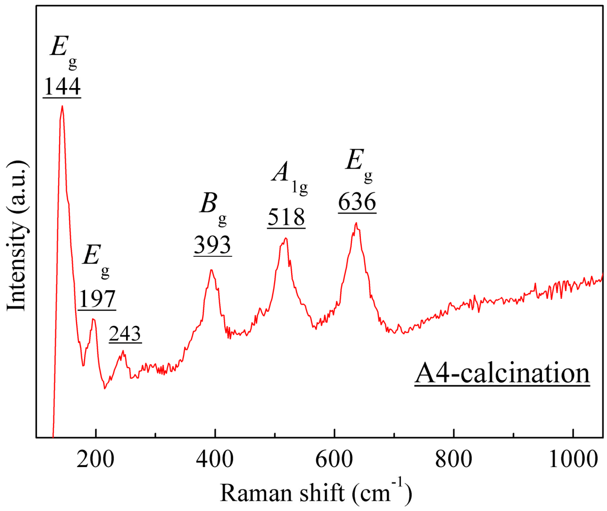

Figure S2. Raman spectrum of the calcined product at 700 °C of the solid sample A4 from washing natisite with water and sulfuric acid solutions.

2. Crystal Information Files

natisite.cif—Crystal Information File of natisite (Na_2TiSiO_5) optimized with CA-PZ/830 eV.

H_2TiSiO_5.cif—Crystal Information File of the intermediate structure (H_2TiSiO_5) obtained from washing natisite with water sulfuric acid solutions.

3. Visualization of Photon Modes

natisite_Efield.phonon—The raw vibrational output file for the primitive structure of natisite, delivered by CASTEP computations with LDA/830 eV. All the phonon modes can be directly visualized to describe the atomic displacements of vibrations using freely-available open-source J-Mol* program: http://jmol.sourceforge.net/

*Comment: In order to visualize the attached output with Jmol, one may use the CONSOLE (File/console) with the following commands:

a) load (full path)/ natisite_primitive_Efield.phonon;
b) The left/right arrows at the top select the phonon number;
c) the vibration can be turned ON/OFF with the command 'vibration ON/OFF';

d) its amplitude can be controlled by giving the integer after the command 'vibration SCALE', while the period time scale is controlled using the command 'vibration PERIOD'.
